# Vermiculite’s strong buffer capacity renders it unsuitable for studies of acidity on soybean (*Glycine max* L.) nodulation and growth

**DOI:** 10.1186/1756-0500-6-465

**Published:** 2013-11-14

**Authors:** Arief Indrasumunar, Peter M Gresshoff

**Affiliations:** 1 ARC Centre of Excellence for Integrative Legume Research, and School of Agriculture and Food Sciences, The University of Queensland, St. Lucia 4072, Australia

**Keywords:** Acidity, Buffer, Nodulation, Soybean, Vermiculite

## Abstract

**Background:**

Vermiculite is the most common soil-free growing substrate used for plants in horticultural and scientific studies due to its high water holding capacity. However, some studies are not suitable to be conducted in it. The described experiments aimed to test the suitability of vermiculite to study the effect of acidity on nodulation and growth of soybean (*Glycine max* L.).

**Methods:**

Two different nutrient solutions (Broughton & Dilworth, and modified Herridge nutrient solutions) with or without MES buffer addition were used to irrigate soybean grown on vermiculite growth substrates. The pH of nutrient solutions was adjusted to either pH 4.0 or 7.0 prior its use. The nodulation and vegetative growth of soybean plants were assessed at 3 and 4 weeks after inoculation.

**Results:**

The unsuitability of presumably inert vermiculite as a physical plant growth substrate for studying the effects of acidity on soybean nodulation and plant growth was illustrated. Nodulation and growth of soybean grown in vermiculite were not affected by irrigation with pH-adjusted nutrient solution either at pH 4.0 or 7.0. This was reasonably caused by the ability of vermiculite to neutralise (buffer) the pH of the supplied nutrient solution (pH 2.0 – 7.0).

**Conclusions:**

Due to its buffering capacity, vermiculite cannot be used as growth support to study the effect of acidity on nodulation and plant growth.

## Background

Vermiculite is the mineralogical name given to hydrated laminar magnesium-aluminium-iron silicate ((Mg, Fe, Al)_3_((Al, Si)_4_O_10_)(OH)_2_.4H_2_O). It is an expandable 2:1 mineral and often forms from alteration of mica [1]. It is widely available, easily handled, odourless, and low-cost material [2]. Vermiculite is the most common physical growth substrate used for plants in horticultural and scientific studies due to its high water holding, inert chemical nature, moderate level of aeration, absence of substrate for microbial growth and effective cation-exchange capacities compared to sand or gravel to promote better plant growth.

However, some studies are not suitable to be conducted in this substrate. For example, vermiculite was used as an ‘N-free’ growth medium for the study of associative N_2_-fixation [3], but later research showed that significant quantities of mineral N were released from vermiculite when it was incubated under warm, moist conditions [4]. Marx and Zak [5] found that vermiculite was not suitable for studying the effect of pH on mycorrhizal formation of slash pine (*Pinus elliottii*), as this substrate neutralised the pH of Melin-Norkrans nutrient solutions added to this medium.

Acid soil stress factors have been reported to affect stages of the nodulation process, nitrogen fixation, and plant growth [6-9]. Several types of growth substrates such as liquid (hydroponic) solution, vermiculite, quarzt sand culture, or direct testing of acid soils were commonly used in acid stress studies. The choice of an appropriate growth substrate is very important, because some have the ability to buffer acid treatment. Here we evaluated the properties of vermiculite as plant growth substrate to study its utility for the study of nutrient solution pH on nodulation of soybean.

## Methods

### Nutrient solution preparation

Two nutrient solutions lacking of nitrogen, Broughton and Dilworth [10] and modified Herridge nutrient solution [11] were used to test the effect of acidity stress on soybean grown on vermiculite medium. The pH of nutrient solutions was adjusted to pH 4.0 or 7.0 using 5 N HCl or 5 N NaOH, and each of them was either with or without MES buffer addition. The composition of each nutrient solution is presented in Table 1. In general, Herridge nutrient solution contained higher concentration of each element than B&D nutrient solution. This higher concentration of Herridge medium was expected to have more buffering capacity than B&D plant growth medium.

**Table 1:** Composition of the two media used for studying the effect of acid stress factors on nodulation and growth of soybean

	**Chemical**	**B&D nutrient solution**	**Modified Herridge nutrient solution**
1	CaCl_2_ · 2H_2_0	1000 μM	2500 μM
2	KH_2_P0_4_	500 μM	1000 μM
3	K_2_HPO_4_	-	1000 μM
4	Fe citrate	10 μM	-
5	Fe(III)-EDTA	-	100 μM
6	MgSO_4_ · 7H_2_0	250 μM	2000 μM
7	K_2_SO_4_	1500 μM	-
8	KCl	-	1500 μM
Micro nutrient			
9	MnSO_4_ · H_2_0	1 μM	-
10	MnCl_2_ · 2H_2_O	-	11 μM
11	H_3_BO_3_	2 μM	46 μM
12	ZnSO_4_ · 7H_2_0	0.5 μM	-
13	ZnCl_2_	-	0.8 μM
14	CuSO_4_ · 5H_2_0	0.2 μM	-
15	CuCl_2_ · 2H_2_O	-	0.3 μM
16	CoSO_4_ · 7H_2_0	0.1 μM	-
17	Na_2_MoO_4_ · 2H_2_0	0.1 μM	0.1 μM

The desired pH was adjusted using 5 N HCl or 5 N NaOH.

Depends on the treatment, MES buffer of 20 mM was either added or not to the nutrient solutions.

### Soybean planting and *Bradyrhizobium japonicum* inoculation

Soybean seeds of cv. Bragg were surface-sterilised using 6% of H_2_O_2_ in 70% ethanol for 5 min and rinsed seven times with sterile water. Axenic seeds were planted in sterilised 4 L black plastic pots containing autoclaved vermiculite growth substrate and irrigated with B&D or Herridge nutrient solution at pH 7.0. Plants were grown in a controlled environment glasshouse at 28°/24°C day/night temperatures and 16 h day length for 5 weeks. At day 5, pH treatments commenced by adding B&D or Herridge nutrient solution with or without MES buffer at its corresponding pH to soybean seedlings twice daily. The water availability of the system was kept at field capacity (run-off). At day 7, soybean seedlings were inoculated with a 4-day-old YMB culture of *Bradyrhizobium japonicum* strain CB1809 (10^7^ cell per mL, 5 mL per seedling). Each treatment was replicated three times with four soybean plant placed in each pot. The pH of growth medium was measured in the supernatant suspension of a 1:2.5 (v/v; vermiculite: distilled H_2_O mixture) at day 0, 1, 2 and 3, and week 1, 3 and 4 after inoculation. Uninoculation control was provided to check if there was any contamination. This control was irrigated with B&D or Herridge nutrient solution at pH 7.0 with or without MES addition. Plants were harvested at 3^rd^ and 4^th^ weeks after inoculation for the assessments of nodule number, nodule dry weight, shoot dry weight, and root dry weight. For dry weight measurement, the tissue was placed in an oven at 65°C for 5 d prior to weighing.

### Experimental design and data analysis

The experiment was conducted as a completely randomised design with three replicates. Duncan’s multiple range tests at 1% and 5% probability levels were used for mean separation.

## Results and discussion

### *Bradyrhizobium japonicum* inoculation

**Figure 1:**
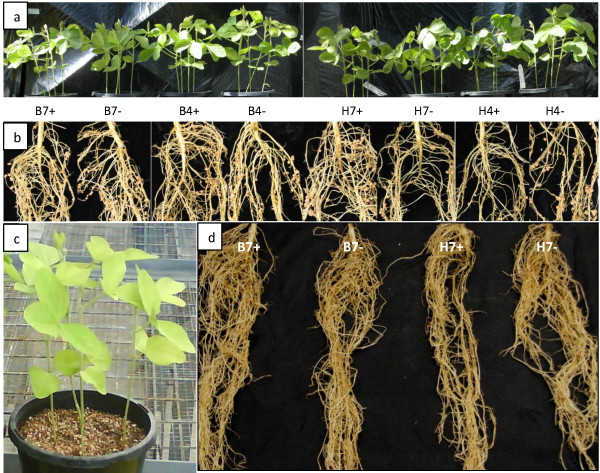
**Four weeks-old inoculated and uninoculated soybean cv Bragg plants grown in glasshouse under controlled conditions.** The plants were watered with nutrient solutions B & D or Herridge with or without MES buffer at pH 4.0 or pH 7.0. Rhizobia inoculation worked well as shown by good shoot growth **(a)** and well nodulated root **(b)**. Uninoculation control was provided to check if there was any contamination; note that growth still occurred from nitrogen stored in the cotyledons **(c)**. Amble growth also seen for roots with large fibrous morphology **(d)**. The length of the shown root system is about 25 cm. B: B&D nutrient solution; H: modified Herridge nutrient solution; 7: pH 7.0; 4: pH 4.0; +: with MES buffer; -: without MES buffer.

*Bradyrhizobium japonicum* inoculation worked well in this experiment (Figure 1) as shown by green leaves and good growth of soybean Bragg (a) and the formation of good and effective nodulation (b) as indicated by pink pigmentation of nodules from expression of rhizobia-associated leghaemoglobin. Contamination was also successfully avoided during the time of this experiment as shown in Figures 1c and d. No nodulation was found in this treatment suggesting that there was no contamination prior and after acid treatment commenced.

### Treatment effects on nodulation and growth of soybean

**Figure 2:**
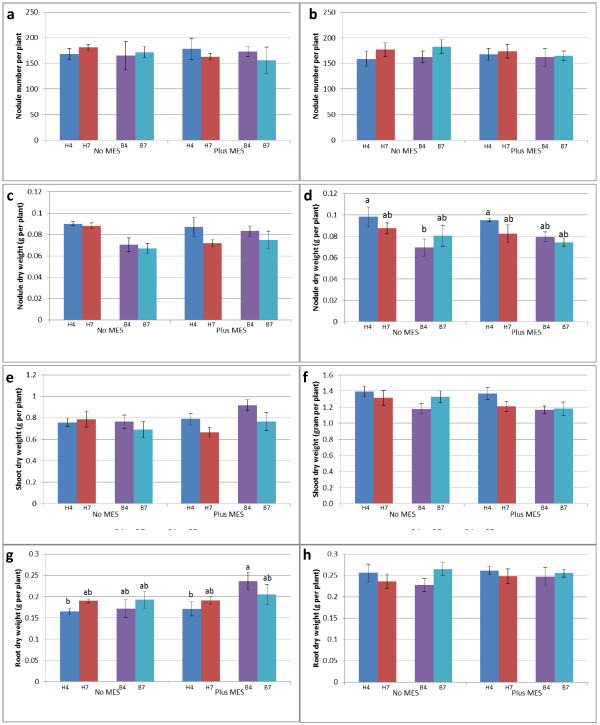
**The effect of different treatments on nodulation and plant growth.** Nodule number per plant at 3 weeks **(a)** and 4 weeks **(b)**; nodule dry weight at 3 weeks **(c)** and 4 weeks **(d)**; shoot dry weight per plant at 3 weeks **(e)** and 4 weeks **(f)**; and root dry weight of soybean (per plant) at 3 weeks **(g)** and 4 weeks **(h)** after inoculation. The data are expressed as means and SD of three replications. B: B&D nutrient solution; H: modified Herridge nutrient solution; 7: pH 7.0; 4: pH 4.0. In each graph, values followed by different letters are significantly (P < 0.05) different.

Nodule number was not affected by all treatments tested (types of nutrient solution, pH of nutrient solutions, or MES buffer addition; Figure 2a,b). Nodule dry weight was also not affected by pH of nutrient solution and MES buffer addition (Figures 2c,d). At week 3, nodule dry weight was not affected by all treatment tested. However, at week 4, soybean watered with B & D nutrient solution (pH 4.0 and ‘No MES’) had less nodule dry weight than soybean watered with Herridge medium of pH 4.0, but not significant to other treatments (Herridge medium pH 7.0, B&D medium pH 4.0 and 7.0). Interestingly, pH level of nutrient solution did not affect nodule number and dry weight at both times of analysis. We also found that there was no increase in nodule number and nodule dry weight from week 3 to week 4 after inoculation. This shows the process of autoregulation of nodulation (AON) [12,13] to maintain the balance of nodule formation.

Shoot dry weight at both harvest times was not affected by any treatment tested (Figure 2e,f). Similarly, pH level did not affect root dry weight of soybean at both harvest times (Figure 2g,h). Other treatments (type of nutrient solution and MES addition) did not affect root dry weight at week 4. However, at week 3, soybean watered with B & D nutrient solution (pH 4.0 + MES) had significantly higher root dry weight than soybean watered with pH 4.0 of Herridge medium, but not to other treatment combinations.

### Effects of MES buffer addition on soybean nodulation and growth

The effects of MES buffer addition on nodulation and growth were measured by comparing the average values of all treatment combinations under MES addition with the average values of all treatment combinations under no-MES addition. We found that addition of MES did not significantly affect nodulation and growth of soybean at both 3 and 4 weeks after inoculation (data not shown). Bugbee and Salisbury [14] also found that MES was biologically inert and does not interact significantly with other ions in solution. They used MES buffer at concentration 1 and 10 mM to test the growth of beans (*Phaseolus vulgaris* L.), maize (*Zea mays* L.), lettuce (*Lactuca sativa* L.), tomatoes (*Lycopersicon esculentum* Mill.), and wheat (*Triticum aestivum* L.). They found that the relative growth rates and plant dry weight among controls and MES treatments were nearly identical for each species during the trial period (3 and 4 weeks). In this experiment we used higher MES concentration (20 mM) to increase the buffering capacity of nutrient solution against high buffering ability of vermiculite. Here we showed that 20 mM MES buffer did not affect nodulation (nodule number and dry weight, both per plant) and growth (shoot and root dry weight) of soybean.

### Effects of nutrient solution types on soybean nodulation and growth

**Figure 3:**
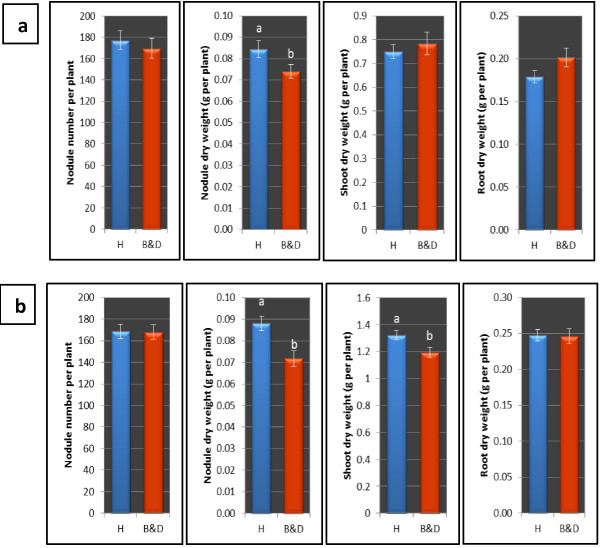
**The effect of nutrient solution types on soybean nodulation and growth at week 3 (a) and week 4 (b) after inoculation.** The data are expressed as means and SD. H: modified Herridge nutrient solution; B&D: Broughton and Dilworth nutrient solution. In each graph, values followed by different letters are significantly (P < 0.05) different. Each value is the mean of 12 data values.

The effects of nutrient solution types on nodulation and growth were measured by comparing the average values of all treatment combinations under B&D with the average values of all treatment combinations under Herridge solution. It shows that at week 3 after inoculation, soybean watered with Herridge medium had significantly higher nodule dry weight than soybean watered with B & D medium (Figure 3a). However, there was no effect on nodule number, shoot dry weight and root dry weight per plant. At week 4, watering with Herridge nutrient solution increased not only nodule dry weight but also shoot dry weight of soybean (Figure 3b). The better nodule development (weeks 3 and 4) and shoot growth (week 4) of plants watered with Herridge medium were caused by the higher, but clearly non-toxic concentration of most elements in this substrate. As shown in Table 1, Herridge nutrient solution contains higher concentration of each element than B&D nutrient solution. The different forms of the elements in Herridge nutrient solution might also determine a different bioavailability of the elements to the plants.

### Effects of nutrient solution pH on soybean nodulation and growth

The effects of nutrient solution pH on nodulation and growth were measured by comparing the average values of all treatment combinations under pH 4.0 with the average values of all treatment combinations under pH 7.0. We found that there was no significant effect of nutrient solution pH on nodulation and growth of soybean at both 3 and 4 weeks after inoculation (data not shown). This is interesting because we expected that nodulation and growth of soybean watered with nutrient solution of pH 4.0 were significantly less than soybean watered with nutrient solution of pH 7.0. Previous experiments also showed that acidity had negative effect on nodulation and growth of soybean [6-8]. To find out the cause of these unexpected results, we analysed the vermiculite properties especially on its effects on pH of the substrate medium.

### Substrate properties

**Figure 4:**
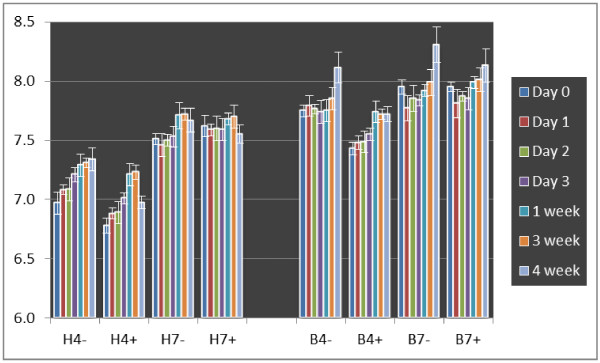
**The change of pH value of vermiculite medium over the period of soybean growth.** The pH was measured based on a 1:2.5 (v/v) suspension in water. B: B&D nutrient solution; H: Herridge nutrient solution; 7: pH 7.0; 4: pH 4.0; +: +MES buffer; -: no MES buffer addition. The data are expressed as means and SD of three replications.

To check the pH values of growth substrate, samples were collected from each pot at days 0, 1, 2 and 3, and 1, 3 and 4 weeks after inoculation (see Figure 4). Here we measured the pH of the bulk vermiculite medium and assume that it corresponds to the pH of the solution around the roots as we did not observe alterations of nodulation and growth using nutrient solutions adjusted to pH 4.0. It is shown here that the real pH of the medium was not similar to the pH of the input nutrient solution. Vermiculite increased each of the tested nutrient solution pH. In general, the pH increase of vermiculite substrate watered with Herridge nutrient solution was lower than vermiculite substrate watered with B&D nutrient solution. This result shows that Herridge medium has higher buffering capacity as a result of its higher concentration of most elements in this nutrient solution (Table 1). The actual pH values of vermiculite substrate watered with pH 4.0 of Herridge and B&D nutrient solutions were around 7.0 and 7.5, respectively. Moreover, the actual pH of vermiculite watered with pH 7.0 of nutrient solutions was around 7.5 and 8.0. We also found that addition of MES buffer into the nutrient solutions was not able to maintain the initial pH of nutrient solutions. It only slightly lowered the pH increase of substrate watered with pH 4.0 nutrient solutions but not to substrate watered with pH 7.0 nutrient solution (Figure 4).

### Vermiculite substrate axenically neutralised nutrient solution pH

**Figure 5:**
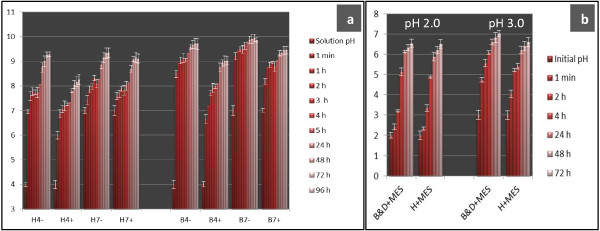
**The change of pH value of the vermiculite after 1 min to 96 hours shaking of the vermiculite suspension.** Initial pH 4.0 and 7.0 **(a)**; initial pH 2.0 and 3.0 **(b)**. B: B&D nutrient solution; H: Herridge nutrient solution; 7: pH 7.0; 4: pH 4.0; +: +MES buffer; -: no MES buffer addition. The data are expressed as means and SD of three replications.

To check the ‘actual’ pH of vermiculite after the addition of nutrient solutions, a simple experiment was conducted. Twenty-five mL of nutrient solution with different pH values was added to 10 mL (2.5 g) of vermiculite. The axenic mixture was shaken (in a Falcon tube; 120 rpm; 28°C) and the pH value of the suspension was measured after 1 min to 96 hours of shaking (Figure 5a). As shown in this figure, vermiculite easily neutralised the pH of both nutrient solutions whether MES buffer was added to the nutrient solution or not. Nutrient solution with MES buffer addition had slightly better buffering capacity than nutrient without MES, but the final pH values were neutral, even alkali in both nutrient solutions. We also found that vermiculite was able to neutralise very acid nutrient solutions (2.0 and 3.0) to around pH 5 and 6 after 4 and 24 hours of shaking, respectively (Figure 5b). From this experiment it is clearly shown that vermiculite has very strong buffering capacity. Only as little as 2.5 g of vermiculite was able to neutralise 25 mL of nutrient solutions with or without MES buffer addition.

Buffering ability of vermiculite was also shown by Duman and Tunç [15] when water of different pH (2.20, 3.0, 5.95, and 10.58) was added to vermiculite substrate. The initial pH value of water was adjusted to 2.20, 3.0, 5.95, and 10.58 by adding HCl or NaOH before preparation of vermiculite/water suspension. Then, vermiculite was added on it and the pH value of the suspension was recorded at various time intervals. Vermiculite increased acid suspension pH from 2.20 to 2.24, from 3.0 to 5.77, from 5.95 to 9.79; and slightly decreased alkaline pH from 10.58 to 10.10 after 180 minutes. Similar to our finding, it is shown here that buffering capacity of vermiculite decreased when the pH of water was very acid (2.20) or very alkali (10.58).

The increase of suspension pH by vermiculite was caused by the adsorption of H^+^ ions from solutions to the negatively charged surface of vermiculite. As explained by Duman and Tunç [2] vermiculite layers have permanent negative charges; but when the pH of solution was very alkali, the transfer of protons from SiOH (or AlOH) groups in the lattice of vermiculite to free OH^-^ ions with formation of H_2_O in the suspension may cause a decrease in pH value.

Similar results were presented long ago by Marx and Zak [5] who found that vermiculite was not suitable for studying the effect of pH on mycorrhizal formation of slash pine (*Pinus elliottii*). Half-century old production schemes still result in similar vermiculite; however, many researchers still using this medium for studying the acidity effects on plant growth. Vermiculite buffered the supplemented nutrient to pH 6.4-6.7 in less than 48 hours, regardless of the original pH (3.0, 3.5, 4.0, 4.5, 5.0, 5.5, 6.0, 6.5, and 6.7); whereas sand and control (nutrient solution) treatments remained reasonably stable. Chilvers [15] reported that vermiculite brought about a rapid rise in pH because of its high cation exchange capacity (CEC). Cation Exchange Capacity is the total capacity of soil to hold exchangeable cations. It is an inherent soil characteristic and is difficult to alter significantly. It influences the soil’s ability to hold onto essential nutrients and provides a buffer against soil acidification. The CEC of vermiculite was 105–174 meq/100 grams [16].

To counteract the buffering action of vermiculite, Mark and Zak [5] mixed vermiculite with small quantities of finely ground peat moss. A ratio of 20 mL of peat moss to 880 mL of vermiculite produced a pH of 6.0; and a ratio of 120:780 produced a pH of 4.0. The pH decreased proportionately as the volume of peat moss was increased. The mixture of vermiculite and peat moss established a stable pH for the length of the experiment (4 months).

Recently, Manassila et al. [17] also used vermiculite to test the effect of acidity on soybean nodulation by *Bradyrhizobium* USDA110 and acid tolerance *Bradyrhizobium* sp. DASA01007. They tried to modify the pH of vermiculite by soaking it in buffered nutrient solution at different pH (pH 4.5 or 6.8) 24 h before planting. The pHs of vermiculite was maintained by adding the desired pH of buffered nutrient solution during the experiment. They also found that acid treatment (pH 4.5 vs pH 6.8) did not affect nodulation and growth of soybean. However, in their experiment, they did not check the real pH values of the vermiculite medium during the soybean growth. It could be that the real pH of the vermiculite medium was always at around neutrality and was not affected by different pH of supplied nutrient solution.

## Conclusions

The results of this current experiment show that the supplied pH to the vermiculite is not what actually experienced by the plants. Vermiculite neutralised the pH of nutrient solution added to these substrates. The addition of MES buffer into nutrient solution did not help nutrient solution in maintaining its initial pH. Therefore, vermiculite should be cautiously used as growth substrate to study the effect of acidity on nodulation and plant growth. Conversely, there is a positive aspect about vermiculite in that the pH stays constant over a long time, and is not readily altered by acidic or alkaline solutions. By this finding, we expect that in the future, researchers will apply very careful consideration in choosing the right medium for experiments of acidity effects on plant growth.

## Abbreviations

AON: Autoregulation of nodulation; B&D: Broughton and Dilworth; MES: 2-(*N*-morpholino) ethanesulfonic acid; YMB: Yeast manitol broth.

## Competing interests

The authors declare they have no competing interests.

## Authors’ contributions

AI designed, performed and wrote the manuscript. PMG designed and wrote the manuscript. Both authors read and approved the final manuscript.
